# Zr(OH)_4_/GO Nanocomposite for the Degradation of Nerve Agent Soman (GD) in High-Humidity Environments

**DOI:** 10.3390/ma13132954

**Published:** 2020-07-01

**Authors:** Seongon Jang, Dongwon Ka, Hyunsook Jung, Min-Kun Kim, Heesoo Jung, Youngho Jin

**Affiliations:** 4th R&D Institute-6th Directorate Agency for Defense Development, Daejeon 34186, Korea; ondol0809@gmail.com (S.J.); rkehd47@gmail.com (D.K.); junghs@add.re.kr (H.J.); mkkim@add.re.kr (M.-K.K.); hsjung@add.re.kr (H.J.)

**Keywords:** Zr(OH)_4_/GO nanocomposite, graphene oxide, degradation, nerve agent, soman (GD), humidity

## Abstract

Zirconium hydroxide, Zr(OH)_4_ is known to be highly effective for the degradation of chemical nerve agents. Due to the strong interaction force between Zr(OH)_4_ and the adsorbed water, however, Zr(OH)_4_ rapidly loses its activity for nerve agents under high-humidity environments, limiting real-world applications. Here, we report a nanocomposite material of Zr(OH)_4_ and graphene oxide (GO) which showed enhanced stability in humid environments. Zr(OH)_4_/GO nanocomposite was prepared via a dropwise method, resulting in a well-dispersed and embedded GO in Zr(OH)_4_ nanocomposite. The nitrogen (N_2_) isotherm analysis showed that the pore structure of Zr(OH)_4_/GO nanocomposite is heterogeneous, and its meso-porosity increased from 0.050 to 0.251 cm^3^/g, compared with pristine Zr(OH)_4_ prepared. Notably, the composite material showed a better performance for nerve agent soman (GD) degradation hydrolysis under high-humidity air conditions (80% relative humidity) and even in aqueous solution. The soman (GD) degradation by the nanocomposite follows the catalytic reaction with a first-order half-life of 60 min. Water adsorption isotherm analysis and diffuse reflectance infrared Fourier transform (DRIFT) spectra provide direct evidence that the interaction between Zr(OH)_4_ and the adsorbed water is reduced in Zr(OH)_4_/GO nanocomposite, indicating that the active sites of Zr(OH)_4_ for the soman (GD) degradation, such as surface hydroxyl groups are almost available even in high-humidity environments.

## 1. Introduction

Chemical warfare nerve agents including soman (GD) (*O*-pinacolyl methylphosphonofluoridate) are known to be the most lethal among chemical warfare agents (CWAs), have been often used in combat zones as tactical weapons and for terrorism during recent decades [1]. In particular, nerve agents have not only fatal effects in acute phase of poisoning but also considerable long-term complications due to irreversible inhibition of acetylcholine esterase [2]. Although all CWAs are banned by the Chemical Weapons Convention (CWC), several countries are still stockpiling chemical agents [3]. Therefore, effective degradation methods must be prepared in order to protect any important equipment, facilities, and environments from the toxic chemical agents [4,5].

Novel materials making nerve agents decompose into less or nontoxic molecules have been going through noticeable evolutions in recent years [6,7,8,9]. Metal-organic frameworks (MOFs) [10], metal oxides (hydroxides) [11,12], and composite materials [12] have attracted significant attention as potential materials [13,14,15,16]. Zirconium hydroxide (Zr(OH)_4_), which has various surface hydroxyl species and defect sites, is considered as one of the most prominent materials because of its superior sorptive property and wide range of reactivity toward nerve agents [17,18,19,20,21,22]. However, many existing researches on Zr(OH)_4_ studied its effects on simulants, rather than real CWAs [17,20,21,22,23,24]. Furthermore, it is not clearly known how real atmospheric conditions affect its CWAs degradation performance in real-operational field. Many metal oxides and hydroxides are very sensitive to H_2_O and CO_2_ and form byproducts that may block their reactive sites. It is already known that water molecules form additional interfaces on the surface and significantly affect the surface reaction chemistry of metal oxides and hydroxides [25]. 

Graphene oxide (GO), as a derivative of graphene, has a unique 2D lamellar structure and many attractive properties [26]. There are abundant oxygenated functional groups including hydroxyl, epoxy, carboxyl, and carbonyl groups on GO surface, which can bind with the metallic centers of the metal oxides, metal hydroxides, or MOFs. These functional groups can be served as activation sites for the crystal growth onto the surface of GO [27,28]. Moreover, it was previously reported that the GO can improve the catalytic activity by inducing a synergistic effect between components in the composites [29,30]. 

It is known that the GO addition can increase the surface area in the composites with Zr(OH)_4_ due to new pores at the interface of both components in Zr(OH)_4_/GO nanocomposite [20]. Here, we supposed that GO would improve availability of the active sites for reactants to access. In order to benefit from the advantages of both Zr(OH)_4_ and GO, we attempted to synthesize Zr(OH)_4_/GO nanocomposite and pristine Zr(OH)_4_ and identify their degradation abilities toward soman (GD) after exposure to high-humidity air conditions. Zr(OH)_4_/GO nanocomposite and pristine Zr(OH)_4_ were prepared by a dropwise method which is useful to control the rate of precipitation. The degradation of soman (GD) was investigated with the materials after exposure at 80% relative humidity (RH) for various times. The time course of the reactions was studied by phosphorus-31 solid state-magic angle spinning nuclear magnetic resonance (^31^P SS-MAS NMR). Water isotherms and DRIFT analysis were additionally performed to identify the reason for the better ability of Zr(OH)_4_/GO nanocomposite to degrade the soman (GD) agent in high-humidity environments.

## 2. Materials and Methods

### 2.1. Materials

Graphene oxide (GO), zirconium chloride (ZrCl_4_ ≥ 99.5%, powder), and sodium hydroxide (NaOH—reagent grade ≥ 98.0 pellets anhydrous) were purchased from Sigma-Aldrich (Seoul, Korea) and used without any pre-treatment. The soman (GD) agent was synthesized at the Organisation for the Prohibition of Chemical Weapons (OPCW) designated laboratories. The purity of the agent was determined by gas chromatography-mass spectrometry (GC-MS) and hydrogen-1 nuclear magnetic resonance (^1^H NMR) and exceeded 99%.

### 2.2. Preparation of Zr(OH)_4_/GO Nanocomposite and Zr(OH)_4_

**Scheme 1:**
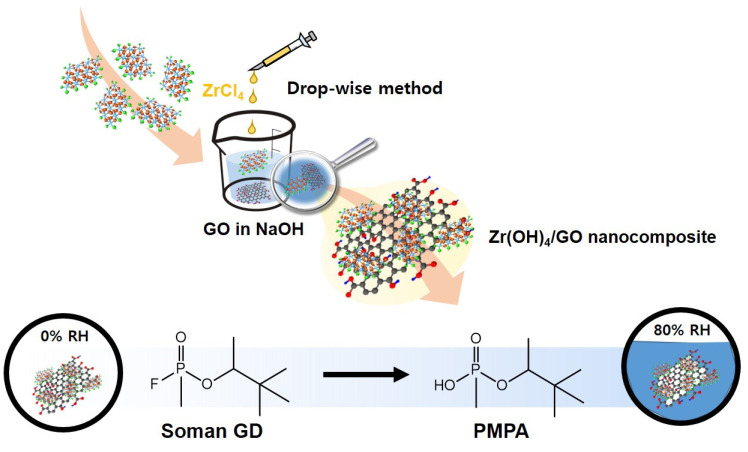
Degradation of nerve agent soman (GD) by Zr(OH)_4_/GO nanocomposite in a high-humidity environment.

NaOH (0.05 M) and ZrCl_4_ (0.05 M) were added into separate containers filled with deionized (DI) water and sonicated for 30 min. GO powder (5 wt % of the final mass of the composite) was dispersed in 1 L of NaOH solution through ultra-sonication for 30 min. A stoichiometric amount of ZrCl_4_ (0.25 L, 0.05 M) solution was dropwise added into the solution at a constant rate (0.75 mL/min) as illustrated in Scheme 1. The suspension was stirred vigorously for 5 h to obtain the precipitate of Zr(OH)_4_/GO nanocomposite. The obtained precipitate was washed several times with deionized water by using a centrifuge at 6000 rpm for 15 min until its pH became nearly neutral (pH 7–8). Finally, it was dried at 60 ℃ in vacuum oven for 48 h. Pristine Zr(OH)_4_ was synthesized in a manner similar to Zr(OH)_4_/GO nanocomposite without the addition of GO.

### 2.3. Characterization

The structures and properties of Zr(OH)_4_ and Zr(OH)_4_/GO nanocomposite materials were characterized by various methods: scanning electron microscopy (SEM) with energy dispersive spectroscopy (EDS), X-ray diffraction (XRD), thermogravimetric analysis (TGA), nitrogen adsorption-desorption isotherms, water vapor adsorption isotherms, and diffuse reflectance infrared Fourier transform spectroscopy (DRIFT).

SEM and EDS were used to analyze the surface morphology and composition of the samples, respectively. The instrument used was a Quanta 650 SEM (FEI, Hillsboro, OR, USA) equipped with EDS detector (X1 Analyzer, EDAX, Berwyn, IL, USA). The materials were coated with gold using a Sputter Coater 108 (Cressington, Oxhey, UK) prior to the SEM observations. The measurements were performed with acceleration voltages ranging from 5 to 15 kV. EDS spectra directly revealed the presence of the atomic elements (Carbon, Oxygen, and Zirconium) in the sample. XRD patterns were obtained using a powder X-ray diffractometer (AXS GmbH, Bruker, Madison, WI, USA), which used CuƘɑ radiation (operated at 40 kV and 40 mA). The diffraction patterns were collected with a 2θ scan from 10° to 70°. TGA measurements were performed to study thermal stability using a thermogravimetric analyzer (TGA Q500, TA Instruments, New Castle, PA, USA). The heating rate was 5 ℃/min and the total N_2_ flow rate was 100 mL/min. The samples were heated up to 800 ℃, starting from 30 ℃. The nitrogen adsorption-desorption isotherms were obtained through an ASAP 2020 system (Micromeritics Instrument Corp., Norcross, GA, USA) at 77 K. The samples were degassed at 393 K under 133 mbar before the measurements. The isotherms were used to calculate the surface area, S_BET_ (BET method); the mesopore volume, V_meso_ (calculated by Barrett–Joyner–Halenda (BJH) method); and the total pore volume, V_t_ (calculated from the last point of the isotherm). To measure DRIFT, a Fourier-transform infrared (FTIR) spectrometer (Thermo Scientific, Nicolet i50, Swedesboro, NJ, USA) was used with the DiffusIR accessory (PIKE Technologies, Fitchburg, MA, USA). Before the measurement, the sample was purged with N_2_ gas to reduce background noise. The spectra were collected 128 times, and the resolution was 0.482 cm^−1^. 

### 2.4. Degradation of Nerve Agent Soman (GD)

Disclaimer: Small doses of the nerve agent soman (GD) are known to be lethal if in contact with the skin or inhaled through the nose or mouth. Experiments should be performed only by trained personnel in adequate facilities.

Zr(OH)_4_/GO nanocomposite and Zr(OH)_4_ (10 mg, each) in a 2 mL vial were investigated with exposure to 80% relative humidity (80% RH) condition at 25 ℃ for various hours (72, 168, and 324 h). Subsequently, 0.4 μL of soman (GD) was added with 20 μL of pentane in each vial containing materials [14]. The vials were agitated vigorously on a vortex mixer for 10 min. The residual GD was extracted with 1.5 mL of ethyl acetate (DAEJUNG, Daejeon, South Korea, Guaranteed Reagent ≥ 99.5%) for 2 h, and the solution was analyzed through GC-MS (MSD 5977A, Agilent Technology, Madison, WI, USA). The column mounted on the GC-MS system was 30 m in length and 250 μm in internal diameter, with a liquid-film thickness of 0.25 μm. The analysis was operated by increasing the temperature from 45 to 230 ℃ at a rate of 10 ℃/min. The carrier gas was helium. The injection volume and total flow were 1 µL and 3 mL/min, respectively. There was no split during the measurement.

### 2.5. Reaction Products Analysis

The degradation product, *O*-pinacolyl-methylphosphonic acid (PMPA) was confirmed by GC-MS (MSD 5977A, Agilent Technology). A portion of the supernatant solution in the glass vial were sampled 10 min after the reaction start, and the sample was sufficiently evaporated at 60 ℃ by N_2_ blowing for 30 min. Prior to analysis, PMPA was derivatized with bis(trimethylsilyl)trifluoroacetamide (Sigma-aldrich, Seoul, Korea, for GC derivatization ≥ 99.0%) at 70 ℃ for 2 h [31,32]. GC-MS analysis showed the trimethylsilyl (TMS) derivative, PMPA-TMS.

### 2.6. ^31^P SS-MAS NMR Analysis

^31^P SS-MAS NMR spectra were obtained using an Oxford NMR instrument (AS600, Agilent Technologies, Santa Clara, CA, USA) equipped with a 4 mm probe using direct polarization, spinning rate of approximately 3000 Hz and a 90° pulse width of 4 μs. Zr(OH)_4_/GO nanocomposite and was packed in the zirconium oxide rotor (46 µL pore volume, Revolution NMR, LLC, Fort Collins, CO, USA) and spiked with 5 μL of soman (GD). The rotor was sealed with Kellogg and Fluoropolymer (Kel-F) caps (Revolution NMR, Fort Collins, CO, USA). The delay time between the pulses was 5 s. The ^31^P SS-MAS NMR spectra were referenced to external phosphoric acid at 0 ppm.

## 3. Results and Discussion

### 3.1. Characterization of Zr(OH)_4_/GO Nanocomposite and Pristine

**Figure 1:**
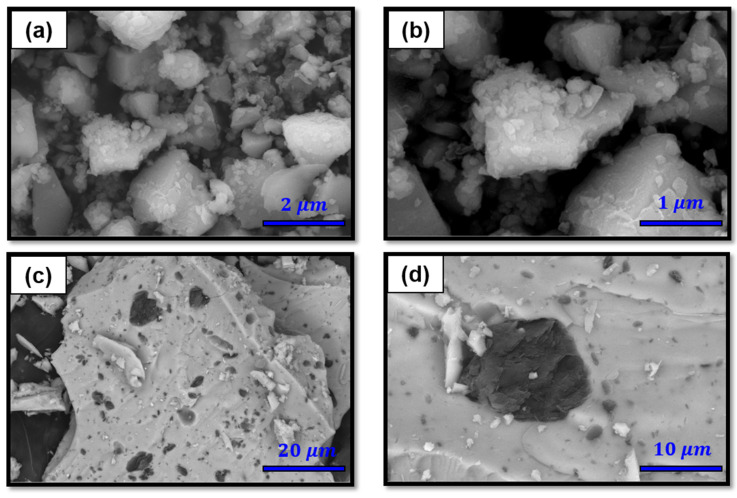
SEM images of Zr(OH)_4_ (**a**) in low and (**b**) in high magnification and Zr(OH)_4_/GO nanocomposite (**c**) in low and (**d**) in high magnification. Images of Zr(OH)_4_/GO nanocomposite were taken in the backscatter mode to distinguish GO from Zr(OH)_4_.

Morphological features and chemical composition of as-synthesized Zr(OH)_4_/GO nanocomposite and pristine Zr(OH)_4_ by dropwise method were characterized by using SEM images and EDS spectra. SEM images of Zr(OH)_4_/GO nanocomposite were taken in the backscatter mode in order to distinguish easily inorganic (Zr(OH)_4_, bright) and organic materials (GO, dark) through the brightness. As presented in Figure 1, many GO flakes were well-dispersed and embedded in Zr(OH)_4_/GO nanocomposite, while small Zr(OH)_4_ particles agglomerated into large particles in pristine Zr(OH)_4_. This uniformity might be attributed to vigorous stirring during the precipitation and the excellent dispensability of GO in aqueous solution. The EDS spectra of Zr(OH)_4_/GO nanocomposite indicates not only that the product consists of C, O, and Zr (Figure S1) but also that GO flakes were successfully introduced on the surface of Zr(OH)_4,_ not just physically mixed. The XRD patterns of Zr(OH)_4_/GO nanocomposite are similar to those of pristine Zr(OH)_4_, which suggests that there was no change in Zr(OH)_4_ structure by forming nanocomposite with GO (Figure S2). Furthermore, both of materials have broad peaks because of their lack of crystallinity.

**Figure 2:**
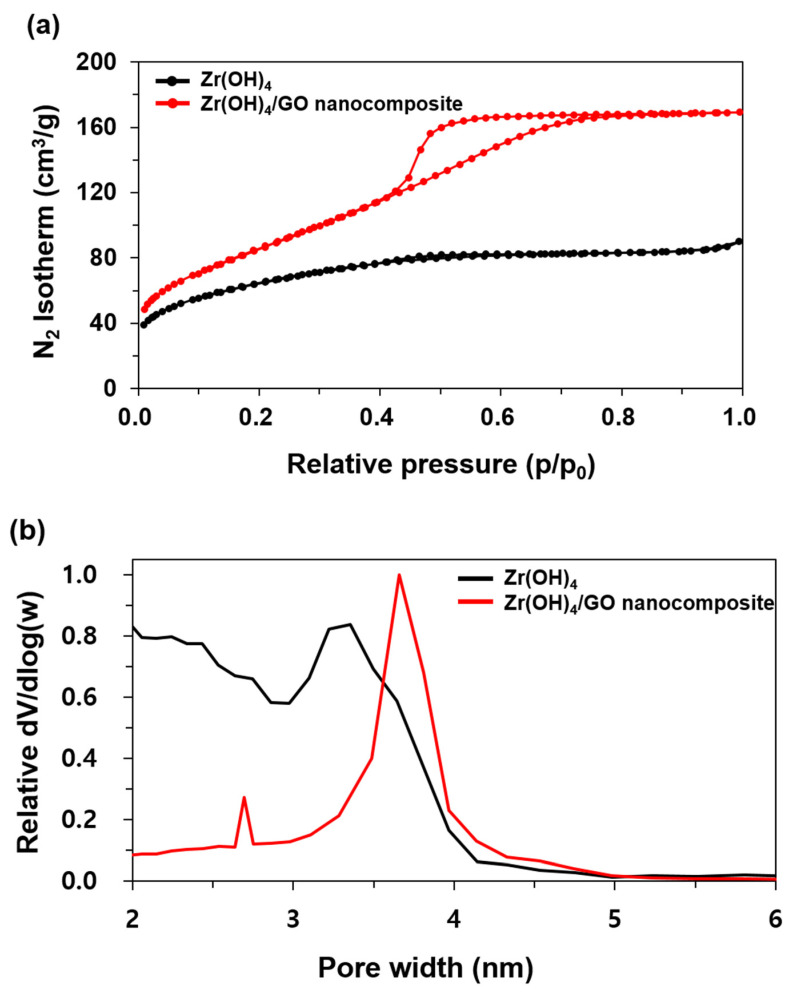
(**a**) N_2_ gas adsorption-desorption isotherms at 77 K and (**b**) pore size distribution for Zr(OH)_4_/GO nanocomposite and pristine Zr(OH)_4_. All samples were degassed at 393 K under 133 mbar before measurement to eliminate impurities in pores. Pore size distribution was analyzed by using the Barrett–Joyner–Halenda (BJH) method based on Kelvin equation.

The nitrogen adsorption-desorption isotherms at 77 K and pore size distribution for Zr(OH)_4_/GO nanocomposite and pristine Zr(OH)_4_ are presented in Figure 2. The Brunauer–Emmett–Teller (BET) surface areas (S_BET_), total pore volumes (V_t_), and mesopore volumes (V_meso_) for Zr(OH)_4_/GO nanocomposite and Zr(OH)_4_ are summarized in Table 1. The nitrogen adsorption–desorption isotherms (Figure 2a) of Zr(OH)_4_/GO nanocomposite showed the higher amount of nitrogen adsorbed and discordance between the nitrogen adsorption and desorption, while pristine Zr(OH)_4_ exhibited typical type I isotherms, as reported in the literature [33]. Indeed, Zr(OH)_4_/GO nanocomposite exhibits a type E hysteresis loop, which is typical characteristic of “ink-bottle” pores [34], corresponding to type IV. The hysteresis loop of Zr(OH)_4_/GO nanocomposite at the relative pressure (p/p_0_) ranging from 0.45 to 0.75 indicates the possible presence of mesoporous structure [35]. The pore size distribution (Figure 2b) demonstrated that most of pores in Zr(OH)_4_/GO nanocomposite were mesopores (2–50 nm in size). The BET surface area increased from 216 to 274 m^2^/g, respectively, in Zr(OH)_4_/GO nanocomposite. It is noted that Zr(OH)_4_/GO nanocomposite has a relatively larger surface area, higher pore volume, and looser structure than that of Zr(OH)_4_. The average pore size also increased from 2–3.2 nm to 3.7 nm after addition of GO particles. These results demonstrate that uniformly embedded GO between Zr(OH)_4_ enhance the structural heterogeneity and porosity of the composite [20], which may improve the adsorption capability of Zr(OH)_4_ [36,37,38].

**Figure 3:**
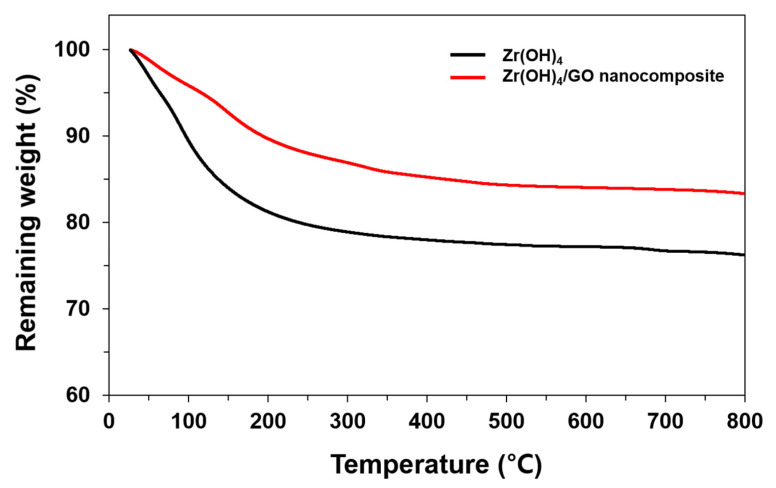
Profiles of thermogravimetric analysis (TGA) of Zr(OH)_4_/GO nanocomposite and pristine Zr(OH)_4_. The loss percentages of Zr(OH)_4_/GO nanocomposite and Zr(OH)_4_ are 17% and 24%, respectively. All samples were heated at 30 to 800 ℃ with a heating rate of 5 ℃/min under N_2_ gas.

The thermal and structural characteristics of Zr(OH)_4_/GO nanocomposite and pristine Zr(OH)_4_ were determined by thermal characterization techniques with elevating temperature. The TGA curves of the materials are presented in Figure 3. Samples were pre-conditioned in the desiccator at ambient temperature for 24 h before TGA analysis. Both of materials showed gradual weight losses in the broad temperature range of 30–500 ℃. The final weight loss at 800 ℃ of Zr(OH)_4_/GO nanocomposite and pristine Zr(OH)_4_ were 14 and 26%, respectively, which were mainly attributed to removal of the physisorbed water and dehydroxylation [20,39]. It should be noted that the weight loss was obviously larger in pristine Zr(OH)_4,_ indicating that more water was adsorbed. These results could be attributed to variation of porosity of Zr(OH)_4_ due to the formation of nanocomposite of GO. 

### 3.2. Hydrolytic Degradation of Soman (GD) by Zr(OH)_4_/GO Nanocomposite and Pristine in High Humidity

**Figure 4:**
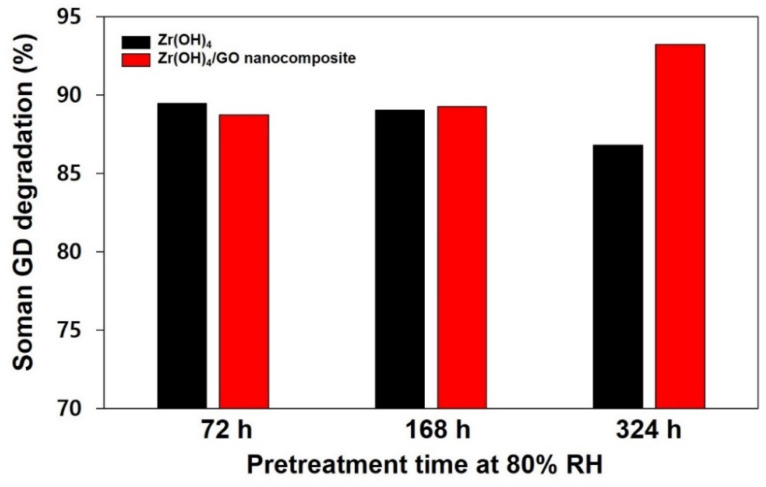
Degradation of nerve agent soman (GD) by and Zr(OH)_4_/GO nanocomposite and pristine Zr(OH)_4_. The samples were pretreated for various times (72, 168, 324 h) at 80% RH in a 25 ℃ convection oven.

To investigate the effect of atmosphere moisture on the degradation of soman (GD) by Zr(OH)_4_/GO nanocomposite and pristine Zr(OH)_4_, the degradation experiments were carried out in the neat state at room temperature. The samples were pretreated for various times (72, 168, and 324 h) at 80% RH in a 25 ℃ convection oven. Figure 4 shows the degradation rate of soman (GD) on Zr(OH)_4_/GO nanocomposite and pristine Zr(OH)_4_ during 10 min reaction time. It is worthy to note that Zr(OH)_4_/GO nanocomposite exhibited better performance for degrading soman (GD) even after 324 h exposure to high-humidity conditions. That is, Zr(OH)_4_/GO nanocomposite maintains a good degradation ability in neat condition whereas Zr(OH)_4_ lost activity for soman (GD) when exposed to 80% RH condition for prolonged time (Figure S3). 

**Figure 5:**
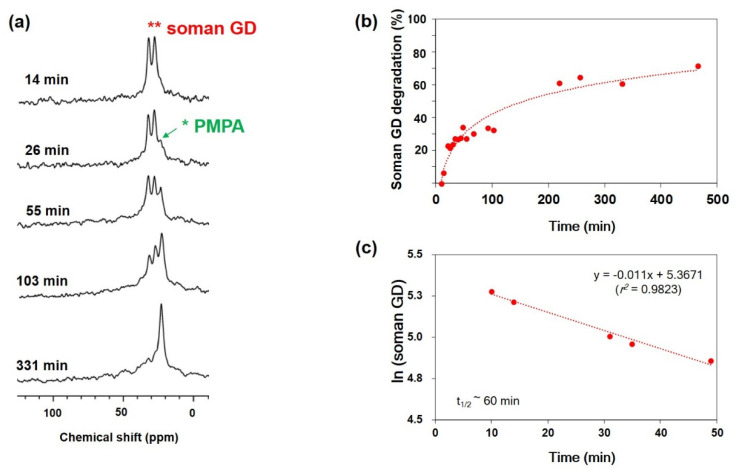
(**a**) ^31^P SS-MAS NMR spectra for the reaction mixture of Zr(OH)_4_/GO nanocomposite and soman (GD), showing the presence of soman (GD) (27.3, 31.6 ppm) and PMPA (24.4 ppm), (**b**) reaction profile of catalytic degradation of soman (GD) upon exposure to Zr(OH)_4_/GO nanocomposite in neat at 25 ℃ soman (GD): 5 μL (0.028 mmol), sample weight: 93.8 mg, and (**c**) the plot of the soman (GD) agent loss in Zr(OH)_4_/GO nanocomposite, showing linearity of the curve with following pseudo-first-order and an estimated half-life of 60 min.

To more clearly confirm the effect of water on the performance of Zr(OH)_4_/GO nanocomposite reactive particles in the degradation reaction of soman (GD), the degradation experiments were performed in aqueous solution. The results, presented in Figure S4, show that the degradation rate of soman (GD) by Zr(OH)_4_/GO nanocomposite in the aqueous solution seems to follow the catalytic reaction, similar to those obtained in previous study [14]. It is noted that the pH of the water containing Zr(OH)_4_/GO nanocomposite is 8.57, which is the same as of pristine Zr(OH)_4_ solution. After reaction with soman (GD), however, the pH of Zr(OH)_4_/GO nanocomposite solution decreased to 5.45, confirming the formation of acidic products, PMPA along with hydrogen fluoride (HF). Although some OH^−^ present by reaction of Zr(OH)_4_/GO nanocomposite with the water may be the hydrolyzing agent for soman (GD), the results, presented in Figure 4 (along with Figure 5 discussed later) show that the hydrolysis is more directly due to Zr(OH)_4_/GO nanocomposite rather than the pH itself of the aqueous solution. 

Additionally, ^31^P SS-MAS NMR was used to determine the resulting reaction rates of soman (GD) on Zr(OH)_4_/GO nanocomposite. As shown in Figure 5, over time, the sharp, twin peaks for soman (GD) at 27.3 and 31.6 ppm are replaced by a broad, single peak for PMPA near 24.4 ppm with its attendant spinning sidebands. The reaction profile shown in Figure 5b clearly shows the hydrolysis of soman (GD) by Zr(OH)_4_/GO nanocomposite follows the catalytic reaction as shown in aqueous solution. That is, after a fast, initial reaction, and a steady state is achieved, exhibiting a first-order half-life of 60 min. By comparison, the half-lives observed for the soman (GD) reaction have been reported as 8.7 min by Zr(OH)_4_, 28 min by MgO, and 270 min by CaO, each [18,40]. Catalytic turnover frequency (TOF) of Zr(OH)_4_/GO nanocomposite in the solid state environment was estimated as 0.0005 min^−1^ by dividing the initial rate (in mmole·min^−1^) by the catalyst loading (in mmoles) [41]. Overall, the reaction of soman (GD) with Zr(OH)_4_/GO nanocomposite is consistent with that previously reported for Zr(OH)_4_ and other solid hydrolysis catalysts [18,19]. From the above results in neat and aqueous solutions, it can be seen that Zr(OH)_4_/GO nanocomposite, unlike Zr(OH)_4_ itself, is suitable for decomposing nerve agents regardless of the air humidity conditions and even in the aqueous solution.

The degradation of soman (GD) is reportedly known to generate a nontoxic product, PMPA which has been shown to reside as surface-bound complexes [18,42,43]. The soman (GD) agent mainly degraded to PMPA upon reaction with Zr(OH)_4_/GO nanocomposite as well (Scheme 1, Figure 5 and Figure S5). No further hydrolysis of PMPA to methylphosphonic acid (MPA) was observed during the 24 h observation period. Although soman (GD) can be hydrolyzed by surface hydroxyl groups and/or physisorbed water on the nanocomposite, we believe, the active hydroxyl sites are probably more available in Zr(OH)_4_/GO nanocomposite for the soman (GD) degradation.

### 3.3. Discussion of Water Adsorption Isotherms and DRIFT Spectra of Zr(OH)_4_/GO Nanocomposite and Pristine Zr(OH)_4_

**Figure 6:**
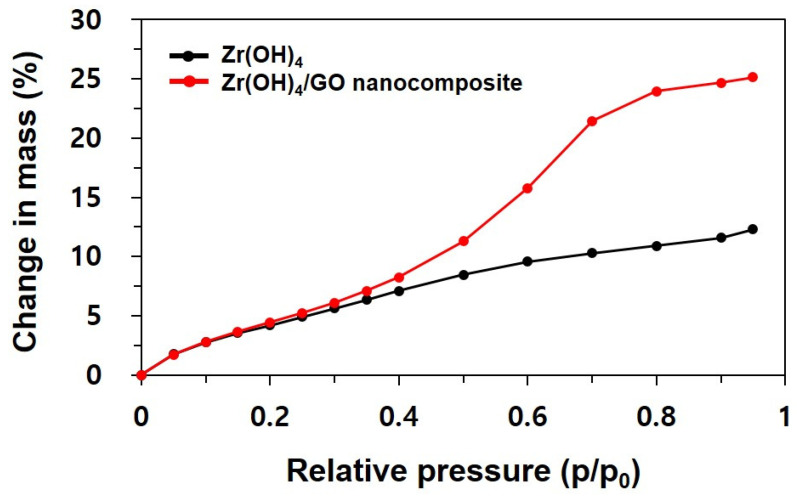
Water adsorption isotherms of Zr(OH)_4_/GO nanocomposite and pristine Zr(OH)_4_ at 25 ℃.

To investigate the reason for the differences in the soman (GD) agent hydrolysis of the materials in wet atmosphere conditions, water adsorption isotherms and DRIFT spectra were obtained for both Zr(OH)_4_/GO nanocomposite and pristine Zr(OH)_4_. First of all, water adsorption isotherms were obtained to examine the interaction between the materials and water. As shown in Figure 6, Zr(OH)_4_/GO nanocomposite exhibits type IV water isotherm, whereas Zr(OH)_4_ shows type I water isotherm, as defined by International Union of Pure and Applied Chemistry (IUPAC) classification [16,44]. The type I water adsorption isotherm is characteristic of a hydrophilic material [14,45]. On the other hand, the type IV isotherm showing a sigmoidal course of an adsorption isotherm is usually observed the adsorption of water on hydrophobic porous materials such as aluminum phosphate, zeolite, metal–organic frameworks, and activated carbon [46]. Therefore, it could be speculated that the active sites of Zr(OH)_4_/GO nanocomposite are more available than those of Zr(OH)_4_, and, as a result, the soman (GD) interacts with more active sites, such that the soman (GD) hydrolysis ability of Zr(OH)_4_ could be enhanced even under atmosphere water present at 80% RH.

**Figure 7:**
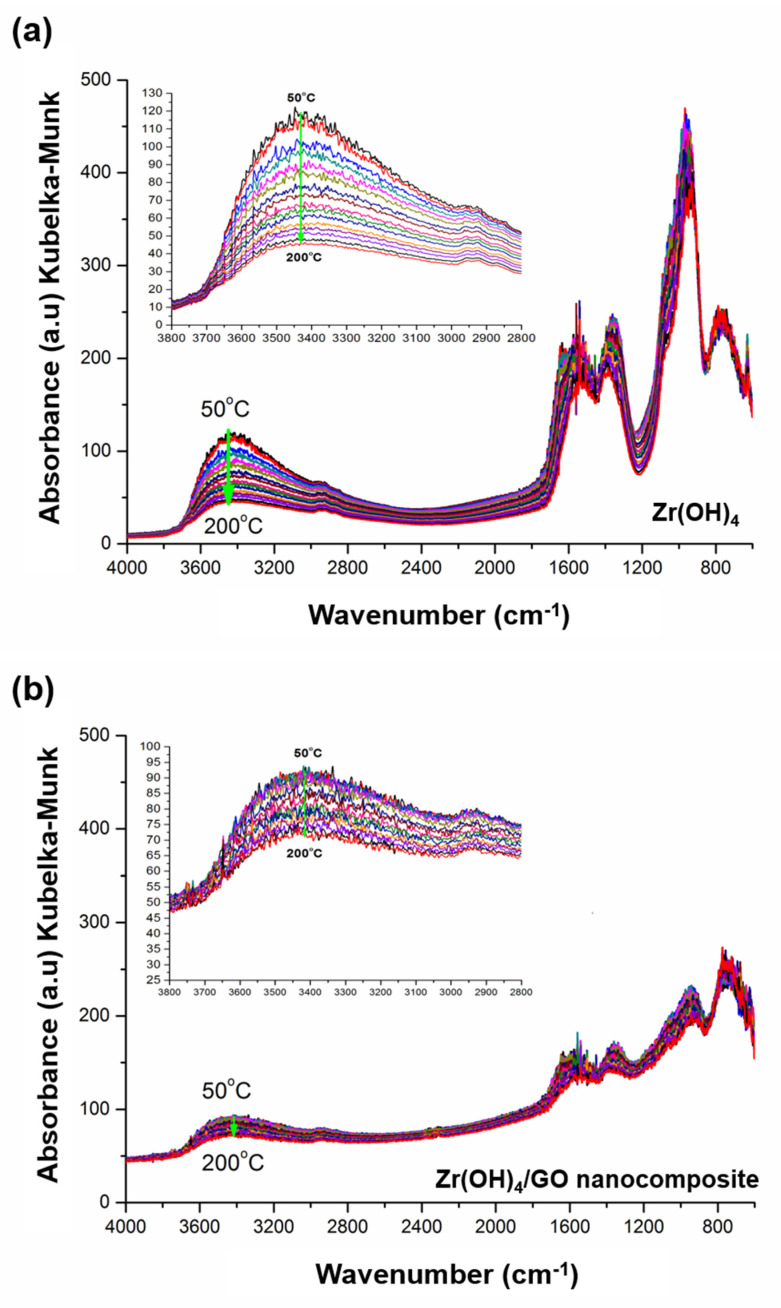
Time-resolved absorbance DRIFT spectra of (**a**) Zr(OH)_4_/GO nanocomposite and (**b**) pristine Zr(OH)_4_. The temperature of the sample cup for DRIFT increased from 50 to 200 ℃ at a rate of 10 ℃/min and was held for 5 min after every 10 ℃ increase. Spectra were collected every 5 min.

DRIFT spectra were obtained to more directly confirm the interaction between the materials and the adsorbed water. Both Zr(OH)_4_/GO nanocomposite and pristine, Zr(OH)_4_ were pretreated at 25 ℃ and 80% RH in a convection oven overnight before the DRIFT measurements. The DRIFT spectra presented in Figure 7 show how the water adsorbed on the surface of the materials changes with increasing temperature (from 50 to 200 ℃). For both materials, the broad peaks from 2800 to 3600 cm^−1^, corresponding to the hydrogen-bonded water (adsorbed) stretching, decrease with increasing temperature by being desorbed. However, it is worth noting that the decrease of this band was considerably greater for pristine Zr(OH)_4_ than for the Zr(OH)_4_/GO nanocomposite, indicating that the interaction between the material and the adsorbed water is weaker in Zr(OH)_4_/GO nanocomposite than that of Zr(OH)_4_. These results confirm that the more active sites such as the surface hydroxyl group are available for the degradation of soman (GD) in the case of Zr(OH)_4_/GO nanocomposite exposed to a humidity environment of 80% RH.

It is worthy to note that GO in the composite with Zr(OH)_4_ can make the pores less hydrophilic, and thus prevent water from blocking the free hydroxyl groups of the surface. We believe that in the case of Zr(OH)_4_/GO nanocomposite, the increase in mesoporous spaces and in hydrophobicity generated certain synergy, resulting in the better hydrolytic performance of soman (GD) after the long-term exposure to the high-humidity environment, such as 80% RH. 

## 4. Conclusions

Zr(OH)_4_/GO nanocomposite and pristine Zr(OH)_4_ were prepared by a simple dropwise method. In particular, Zr(OH)_4_/GO nanocomposite demonstrated enhanced performance for the degradation of the soman (GD) agent after a long exposure under high-humidity air conditions. The characterization studies showed that GO are well-embedded in Zr(OH)_4_/GO nanocomposite, displaying a more porous structure and larger specific surface areas than pristine Zr(OH)_4_. Reaction profiles of Zr(OH)_4_/GO nanocomposite with soman (GD) show that after a fast initial reaction, a steady state is achieved exhibiting a first-order half-life of 60 min and TOF of 0.0005 min^−1^. The water adsorption and DRIFT data of Zr(OH)_4_/GO nanocomposite showed that the interaction between the materials and the adsorbed water was weaker in Zr(OH)_4_/GO nanocomposite, indicating that soman (GD) will have more access to the active sites in the high-humidity condition. With further optimization, the Zr(OH)_4_/GO nanocomposite has the potential to be used in the real world for decontamination application due to superior properties.

## Figures and Tables

**Table 1:** Characteristics of the porous structure of and Zr(OH)_4_/GO nanocomposite and pristine Zr(OH)_4_.

Sample	S_BET_ (m^2^/g)	V_T_ (cm^3^/g)	V_meso_ (cm^3^/g)	V_meso_/V_T_
Zr(OH)_4_	216	0.139	0.050	0.640
Zr(OH)_4_/GO Nanocomposite	274	0.262	0.251	0.958

## Supplementary Materials

The following are available online at https://www.mdpi.com/1996-1944/13/13/2954/s1, Figure S1: (a) SEM images and EDS spectral results of the elements C, O, Zr of Zr(OH)_4_/GO nanocomposite (b) Spot 1, (c) Spot 2, Figure S2: XRD patterns for pristine Zr(OH)_4_ and Zr(OH)_4_/GO nanocomposite. XRD patterns were obtained from 10° to 70°, indicating that there is no change in Zr(OH)_4_ structure by forming nanocomposite with GO, Figure S3: (a) GC of remaining soman (GD) after reaction with Zr(OH)_4_/GO nanocomposite and Zr(OH)_4_, each under 80% RH pretreatment for 324 h. GC spectrum of soman (GD) itself was inserted for comparison, (b) enlarged spectra of GC in (a) for clarity, Figure S4: Comparison of hydrolytic degradation of the nerve agent, soman (GD) by exposure to Zr(OH)_4_/GO nanocomposite and pristine Zr(OH)_4_ in the water at ambient temperature, Figure S5: (a) GC spectra of soman (GD) standard (*R_t_* = 11.7, 11.8 min) and PMPA-TMS (*R_t_* = 15.9 min). Mass spectra of (b) soman (GD) and (c) PMPA-TMS. Note the characteristic fragment ion peaks were seen: m/z = 99.0, 126.1 for GD and m/z = 153.0, 169.1 for PMPA-TMS.
