# Supplementary material for: Zr(OH)4/GO Nanocomposite for the Degradation of Nerve Agent Soman (GD) in High-Humidity Environments
Source: Materials (Basel). 2020 Jul 1;13(13):2954. doi: 10.3390/ma13132954 (PMC7372395; doi:10.3390/ma13132954)
Supplement: Supplementary file 1 [file materials-13-02954-s001.pdf]

materials

Supplementary Materials: $Zr(OH)_4$/GO Nanocomposite for the Degradation of Nerve Agent Soman (GD) in High-Humidity Environments

Seongon Jang †, Dongwon Ka †, Hyunsook Jung, Min-Kun Kim, Heesoo Jung and Youngho Jin *

4th R&D Institute-6th Directorate Agency for Defense Development, Daejeon 34186, South Korea; ondol0809@gmail.com (S.J.); rkehd47@gmail.com (D.K.); junghs@add.re.kr (Hy.J.); mkkim@add.re.kr (M.-K.K.); hsjung@add.re.kr (He.J.)

* Correspondence: cadetnet@add.re.kr; Tel.: +82-42-821-2203; Fax: +82-42-823-3400

S1. Scanning Electron Microscope (SEM)

SEM image and EDS were taken with Quanta 650 SEM (FEI) equipped with EDS detector (X1 Analyzer, EDAX). Sample was sputtered with gold layer to increase the electro-conductivity of surface. Working distances between sample and detector were within 10–12 mm and set voltage was 10 keV.

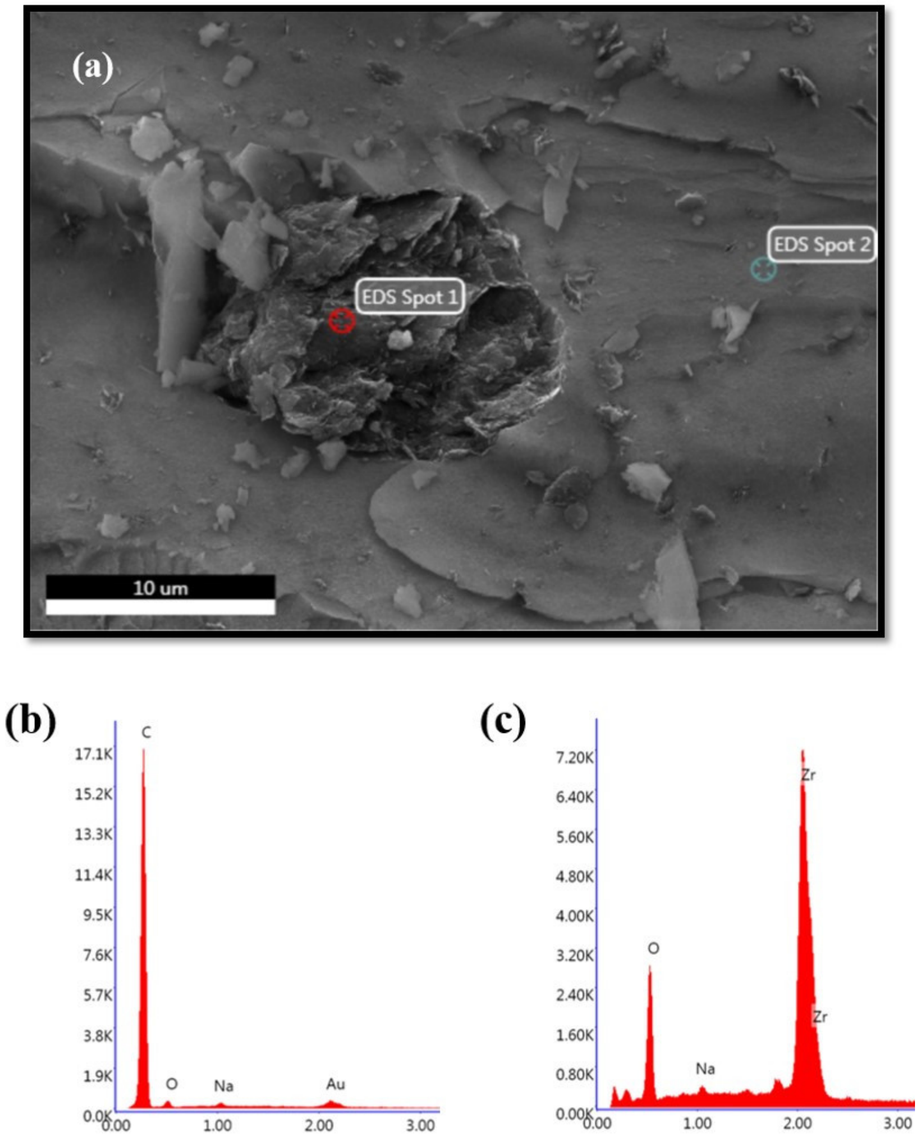

Figure S1. (**a**) SEM images and EDS spectral results of the elements C, O, Zr of $Zr(OH)_4$/GO nanocomposite (**b**) Spot 1, (**c**) Spot 2.

S2. X-ray Diffraction (XRD) Patterns

XRD patterns were obtained using a AXS GmbH powder X-ray diffractometer (Bruker), which used CuKα radiation (operated at 40 kV and 40 mA). The diffraction patterns were collected with a 2θ scan from 10° to 70°.

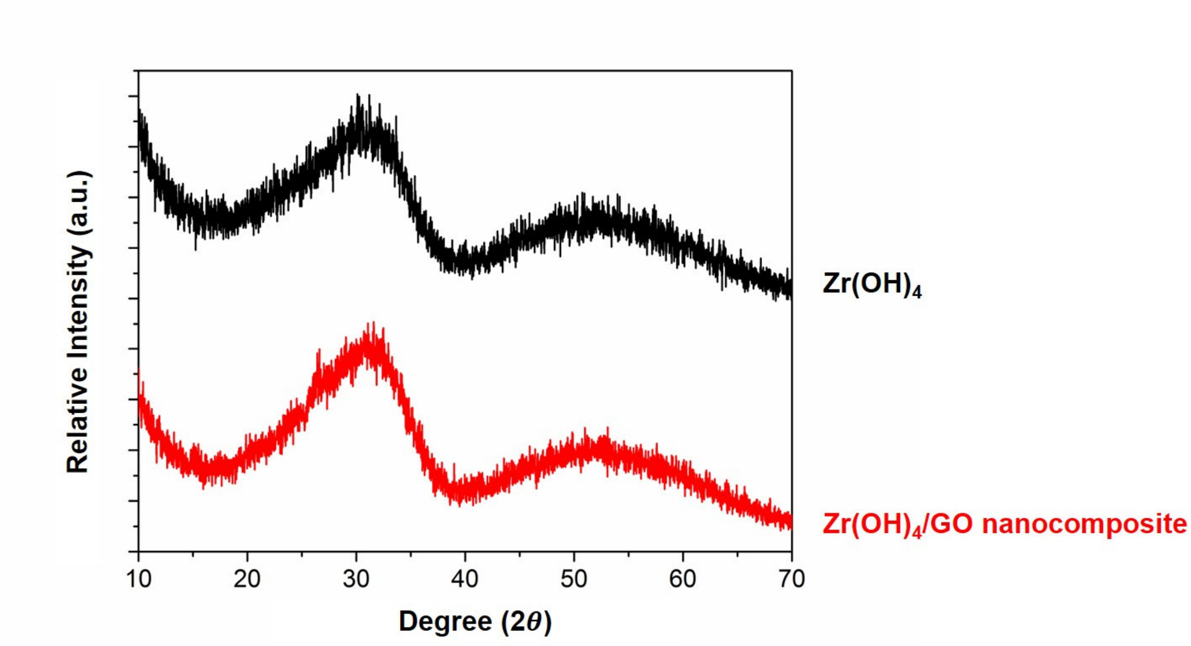

Figure S2. XRD patterns for pristine $Zr(OH)_4$ and $Zr(OH)_4$/GO nanocomposite. XRD patterns were obtained from 10° to 70°, indicating that there is no change in $Zr(OH)_4$ structure by forming nanocomposite with GO.

S3. GD Degradation in Neat Condition

0.4 µL of soman (GD) and 20 µL of pentane were added in glass vials containing $Zr(OH)_4$ and $Zr(OH)_4$/GO nanocomposite, each 10 mg. $Zr(OH)_4$/GO nanocomposite and $Zr(OH)_4$ were exposed to 80% RH condition for 324 h prior to reaction with GD. The vials containing reaction mixtures were agitated for 2 min on vortex mixer and left standing at room temperature. Each vial was taken out for analysis at 10 min reaction. The residual soman (GD) agent was extracted in 1.5 mL of ethyl acetate for 2 h and the solution was subjected to GC analysis.

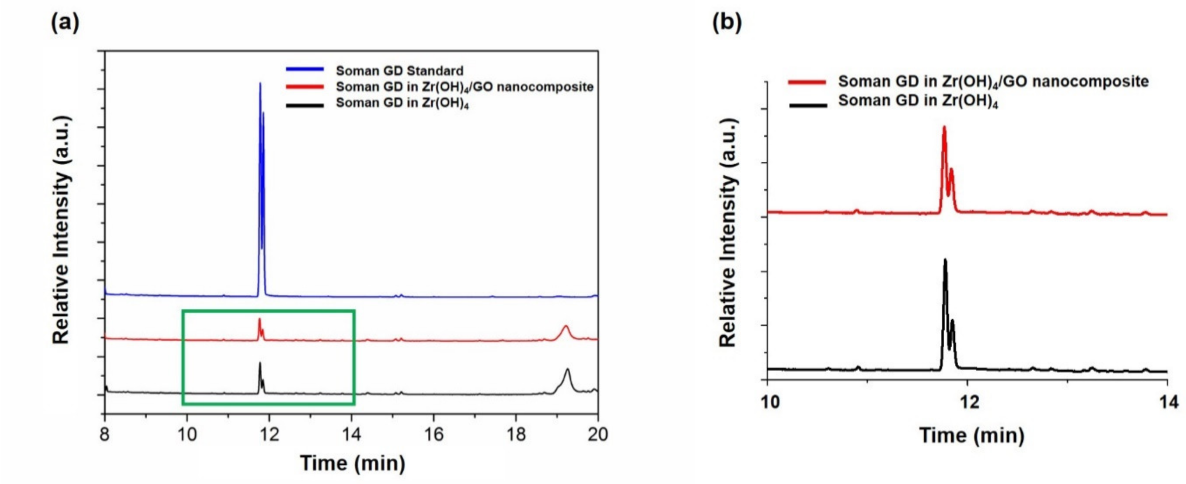

Figure S3. (**a**) GC of remaining soman (GD) after reaction with $Zr(OH)_4$/GO nanocomposite and $Zr(OH)_4$, each under 80% RH pretreatment for 324 h. GC spectrum of soman (GD) itself was inserted for comparison, (**b**) enlarged spectra of GC in (a) for clarity.

S4. Profiles of Hydrolytic Degradation of Soman (GD) Upon Exposure to the $Zr(OH)_4$/GO Nanocomposite

The degradation of soman (GD) (2.5 μL, 0.014 mmol) was studied suspending 20 mg of $Zr(OH)_4$/GO nanocomposite and pristine $Zr(OH)_4$, respectively, in 0.5 mL of a mixture, H_2O (distilled water, 0.25 mL) and acetronitrile (Sigma-Aldrich, for HLPC, ≥ 99.9%, 0.25 mL). 1.5 μL of cyclohexanol (Sigma-Aldrich, Reagent Plus, 99%) was added as internal standard. The mixture was agitated for 1 min. The evolution of the concentration of soman (GD) was followed at room temperature by means of GC, employing a flame ionization detector (GC-FID 6850, Agilent), HP-5, 30 m-column (0.25 mm internal diameter) and taking 0.1 μL aliquots of the supernatant solution.

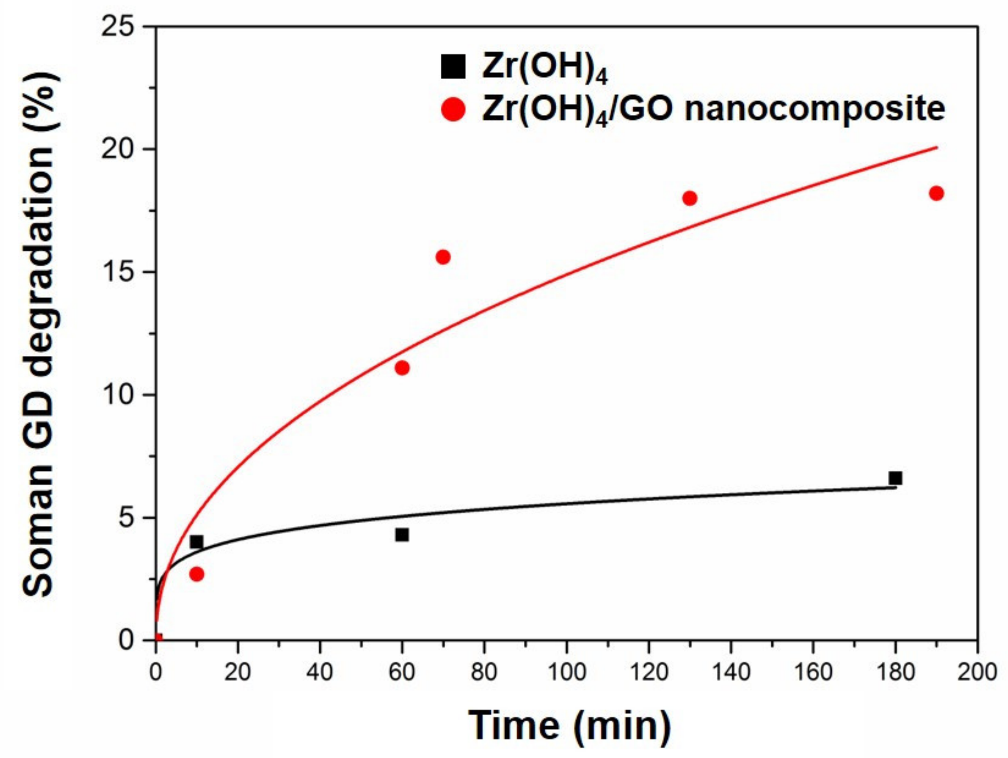

Figure S4. Comparison of hydrolytic degradation of the nerve agent, soman (GD) by exposure to $Zr(OH)_4$/GO nanocomposite and pristine $Zr(OH)_4$ in the water at ambient temperature.

S5. Reaction Products Analysis

A degradation product of soman (GD) by $Zr(OH)_4$/GO nanocomposite was confirmed by GC-MS. A portion of the supernatant solution in the glass vial were sampled 2 h after the reaction start, and the sample was sufficiently evaporated at 60 °C by N_2 gas blowing. After the silylation process, GC-MS analysis showed that the major byproduct was trimethylsilyl (TMS) derivative of *O*-pinacolyl methyl phosphonic acid (PMPA-TMS) for soman (GD) agent hydrolysis by $Zr(OH)_4$/GO nanocomposite.

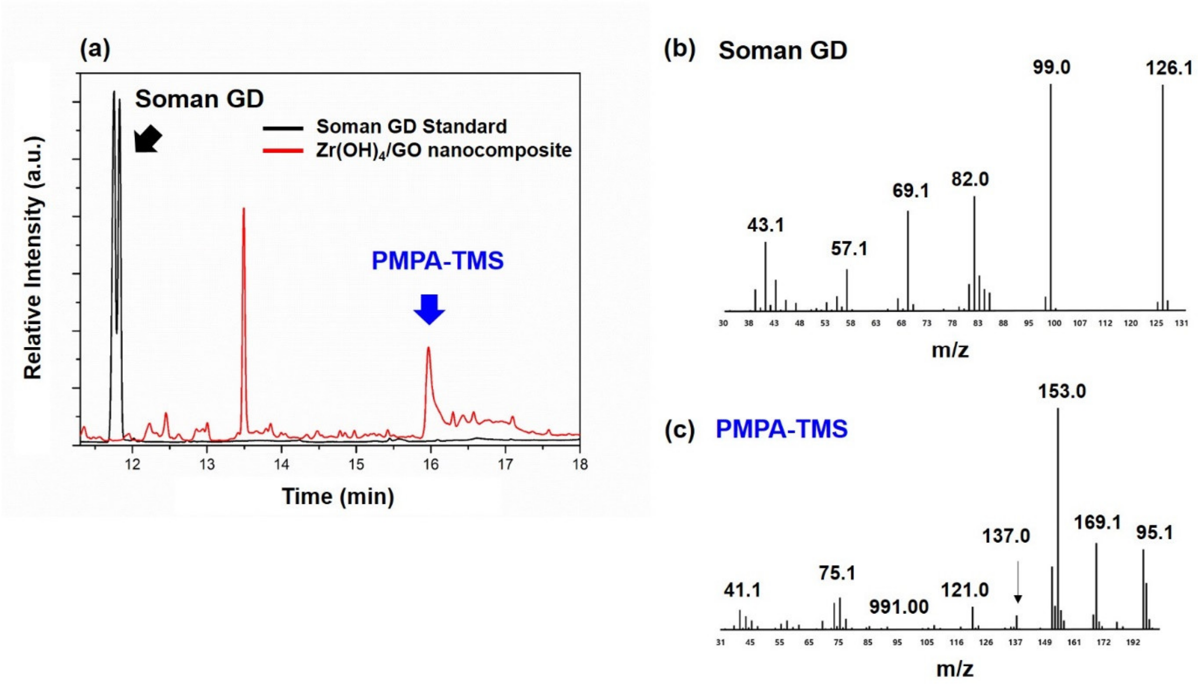

Figure S5. (**a**) GC spectra of soman (GD) standard (R_t = 11.7, 11.8 min) and PMPA-TMS (R_t = 15.9 min). Mass spectra of (**b**) soman (GD) and (**c**) PMPA-TMS. Note the characteristic fragment ion peaks were seen: m/z = 99.0, 126.1 for GD and m/z = 153.0, 169.1 for PMPA-TMS.

S6. pH Measurement of the Reactive Materials Solutions.

120 mg of each reactive material ($Zr(OH)_4$/GO nanocomposite, pristine $Zr(OH)_4$) was placed in 3 mL of deionized water and allowed to stand for 3 h prior to the pH measurements. After the reaction with 15 μL of soman (GD) for 1.5 h, pH was measured as well. The pH meter used was Orion Versa Star Pro of Thermo Scientific Co. and the electrode was ROSS ultra pH/ATC Triode (8175BNUMD).
